# Starting Age for Screening Metabolic Dysfunction‐Associated Steatotic Liver Disease in Children Using Controlled Attenuation Parameter on Transient Elastography

**DOI:** 10.1111/ijpo.70080

**Published:** 2025-12-22

**Authors:** Li‐Wen Lee, Jrhau Lung, Ju‐Bei Yen, Chao‐Yu Chen, Yu‐San Liao

**Affiliations:** ^1^ Department of Diagnostic Radiology Chang Gung Memorial Hospital Chiayi Taiwan; ^2^ School of Medicine, College of Medicine Chang Gung University Taoyuan Taiwan; ^3^ Department of Medical Research and Development Chang Gung Memorial Hospital Chiayi Taiwan; ^4^ General Education Center Chang Gung University of Science and Technology Chiayi Taiwan; ^5^ Department of Pediatrics Chang Gung Memorial Hospital Chiayi Taiwan; ^6^ School of Traditional Chinese Medicine, College of Medicine Chang Gung University Taoyuan Taiwan; ^7^ Department of Obstetrics and Gynecology Chang Gung Memorial Hospital Chiayi Taiwan; ^8^ Graduate Institute of Clinical Medical Sciences, College of Medicine Chang Gung University Taoyuan Taiwan; ^9^ Department of Diagnostic Radiology Chang Gung Memorial Hospital Yunlin Taiwan

**Keywords:** fatty liver disease, paediatric obesity, vibration controlled transient elastography

## Abstract

**Background:**

Rising sedentary behaviour and consumption of sugar‐sweetened beverages and ultra‐processed foods have increased the risk and earlier onset of obesity in children.

**Objectives:**

To define controlled attenuation parameter (CAP) cut‐off values in healthy children, assess the prevalence of metabolic dysfunction‐associated steatotic liver disease (MASLD) in children with overweight and obesity, and establish an appropriate starting age for MASLD screening in our population.

**Methods:**

Healthy primary school children were recruited. Hepatic steatosis was assessed using CAP, and body composition data were collected during two periods: August 2020–March 2021 and February 2023–May 2024.

**Results:**

Of 1653 participants (mean age 9.5 ± 1.7 years), 976 children with normal weight were used to establish reference intervals. Hepatic steatosis was defined as a CAP above 247 dB/m (97.5th percentile). The prevalences of MASLD in children with overweight and obesity were 0% and 16%, respectively, in school grade 1, which increased to 19% and 75% in school grade 6 (*p* < 0.001).

**Conclusions:**

Screening for hepatic steatosis associated with obesity should begin as early as school grade 1, while screening for children with overweight may be more appropriate by school grade 6. These findings emphasise the need for age‐ and weight‐specific screening strategies for children with overweight and obesity.

**Abbreviations**
ALT: alanine aminotransferase
CAP: controlled attenuation parameter
LSM: liver stiffness measurement
MASLD: metabolic dysfunction‐associated steatotic liver disease
SLD: steatotic liver disease

## 1 Introduction

Metabolic dysfunction‐associated steatotic liver disease (MASLD), the updated term for non‐alcoholic fatty liver disease, is defined by the presence of steatotic liver disease (SLD), diagnosed with imaging or biopsy, along with at least one cardiometabolic risk factor, in the absence of other identifiable causes of hepatic steatosis [1]. Given that overweight or obesity is a recognised cardiometabolic risk factor, children with SLD who fall within the BMI categories of overweight or obesity are diagnosed with MASLD. MASLD is the most common liver disease among children. A meta‐analysis published in 2015, which included studies conducted before October 2013, estimated the global prevalence of SLD in the general paediatric population to be 7.6% [2]. However, a more recent meta‐analysis published in 2024, covering studies from January 1997 to April 2023, reported a global increased prevalence of 13% [3], indicating a rise in paediatric SLD prevalence. Given the varying risk of paediatric SLD across BMI categories—with an incidence of 12% in children with normal weight, 21% in those with overweight, and 47% in those with obesity—screening for SLD is conducted in children with overweight or obesity [3]. This trend highlights the need to reassess whether the currently recommended screening age of 9–10 years is still appropriate [4, 5]. However, studies examining the age‐dependent prevalence of paediatric fatty liver are currently limited.

In children, screening tools for excess fat accumulation in the liver include alanine aminotransferase (ALT) test, imaging or histopathology [1]. SLD can progress or regress through stages, including simple steatosis, steatohepatitis, fibrosis and cirrhosis [6] and therefore, non‐invasive methods—ALT and B‐mode ultrasound are favoured over liver biopsy in clinical practice and epidemiology studies [7]. However, elevated ALT is non‐specific to hepatic steatosis, can be normal in children who have steatosis, and primarily indicates steatohepatitis. In addition, there is a lack of a universal consensus on cut‐off values. Ultrasound is portable, affordable, and widely used, but it relies on operator expertise, cannot assess fibrosis, and struggles to detect mild fatty liver [8]. The ultrasound fatty liver index improves sensitivity slightly (59% to 69%), but overall sensitivity remains low [9]. FibroScan is an ultrasound‐based technique using A‐mode ultrasound for one‐dimensional elastography [10]. It quantifies the controlled attenuation parameter (CAP) for hepatic steatosis and liver stiffness measurement (LSM) for fibrosis [11] and is increasingly studied in children. Although CAP is promising in adults, reference limits for children remain undefined due to limited liver biopsies and insufficient validation studies.

We hypothesise that a reference interval for FibroScan controlled attenuation values can be established from healthy children with normal weight with a low risk of liver disease, setting the threshold for SLD at the 97.5th percentile. This study aimed to establish CAP cut‐off values in healthy children, determine the prevalence of MASLD in children with overweight and obesity, and identify an appropriate starting age to screen for MASLD in our population, using CAP, a diagnostic tool increasingly used to assess SLD.

## 2 Materials and Methods

This prospective cross‐sectional study was performed in line with the principles of the Declaration of Helsinki. Approval was granted by the Institutional Review Board of the Chang Gung Medical Foundation (IRB No: 201901889A3 and 202200380A3). Written informed consent was obtained from all participants and their parents or legal guardians.

### 2.1 Participants

This school‐based study was conducted in two waves: the first from August 2020 to March 2021, which was interrupted by COVID‐19 restrictions, and the second from February 2023 to May 2024, resuming after widespread vaccination to ensure a safer research environment. The study was conducted in the primary schools in Chiayi County, Taiwan, with approval granted for participation in the research. Information and invitation letters were sent to the parents or legal guardians of all students in the participating schools. Eligible participants were healthy children in school grades 1 to 6 who agreed to participate in the study. The exclusion criteria included participants with known liver disease, significant alcohol use, limb defects, pacemaker implants, and chronic illnesses. Participants were excluded if they were absent on the examination day or if they did not complete the body composition analysis or FibroScan test. Each participant underwent basic assessments, body composition measurements, and FibroScan tests at the school health center during the morning session. The time between the FibroScan and the other assessments was no more than 1 week. All measurements were conducted at least 2 h after breakfast.

### 2.2 Body Composition Measurement

Height and weight were measured once, with accuracy to the nearest 0.1 cm and 0.1 kg, respectively, by research assistants. BMI was calculated as the weight (kg) divided by the height squared (m^2^). Age‐ and sex‐specific BMI values were calculated using the World Health Organization (WHO) AnthroPlus software based on the 2007 WHO Reference [12]. Children were categorised based on their weight status as having underweight (BMI *z*‐score < −2SD), normal weight (−2SD ≤ BMI *z*‐score ≤ 1SD), overweight (1SD < BMI *z*‐score ≤ 2SD) or obesity (BMI *z*‐score > 2SD).

Body composition analysis was assessed using a dual‐frequency bioelectrical impedance analyser (Inbody 230, Biospace Corp., Seoul, Korea), validated for school‐aged children by our research group [13]. Fat‐related estimates (body fat mass, trunk fat mass, percentage body fat, and trunk percentage fat) and lean‐related estimates (fat‐free mass and skeletal muscle mass) were measured.

### 2.3 FibroScan Measurements

Before the examination, the children watched a homemade video explaining the FibroScan procedure. They were then positioned in a supine posture, with their right arm under their head, their right leg crossed over the left and instructed to remain motionless. FibroScan measurements were performed using the FibroScan 530 Compact (Echosens, Paris, France) during the first wave and the 430 Mini+ (Echosens, Paris, France) during the second wave. Both FibroScan devices were equipped with M and XL probes, with selection guided by the automated probe selection tool in real time. All measurements were conducted by a single radiologist (LWL). For FibroScan 530, the LSM and CAP measurements were taken simultaneously and considered reliable if at least 10 valid readings were obtained with an interquartile range‐to‐median ratio ≤ 30% for LSM, using the median for data analysis. The FibroScan 430 Mini+, equipped with SmartExam software, used the median of ≥ 10 valid LSM readings for analysis, while its upgraded CAP algorithm required ≥ 200 valid readings, with the mean value used for analysis.

### 2.4 Statistical Analysis

Population reference intervals are typically defined as the central 95% of test results between the 2.5th and 97.5th percentiles, representing the lower and upper limits, respectively [14]. Values above the 97.5th percentile were classified as abnormal [15]. According to the C28‐A3 guideline published by the Clinical and Laboratory Standards Institute and the International Federation of Clinical Chemistry, reference limits should be presented with their 90% confidence intervals, which require at least 120 samples per subgroup to calculate two‐sided 90% confidence intervals [16, 17]. Given the inclusion of children from school grades 1 to 6, a minimum of 720 samples was necessary. The final analysis for CAP and LSM reference intervals included 976 samples, which provided sufficient statistical power and precision to establish accurate reference intervals across different age groups.

Statistical analysis and graphing were conducted using MedCalc Statistical Software version 22.026 (MedCalc Software Ltd., Ostend, Belgium). Outliers were excluded using Tukey's method. Reference intervals were determined using both parametric and non‐parametric methods, in accordance with the C28‐A3 guideline, with 90% confidence intervals for the limits [18]. The normality of the CAP and LSM data was assessed using the Kolmogorov–Smirnov test, with *p* < 0.05 indicating non‐normality. A Box‐Cox transformation with kernel density estimation was applied to transform the data into a symmetric distribution. One‐way ANOVA was used to test the differences in numerical variables across BMI categories, while the frequency of SLD across age groups was analysed using the Cochran‐Armitage trend test.

## 3 Results

### 3.1 Characteristics of the Study Cohort

**FIGURE 1:**
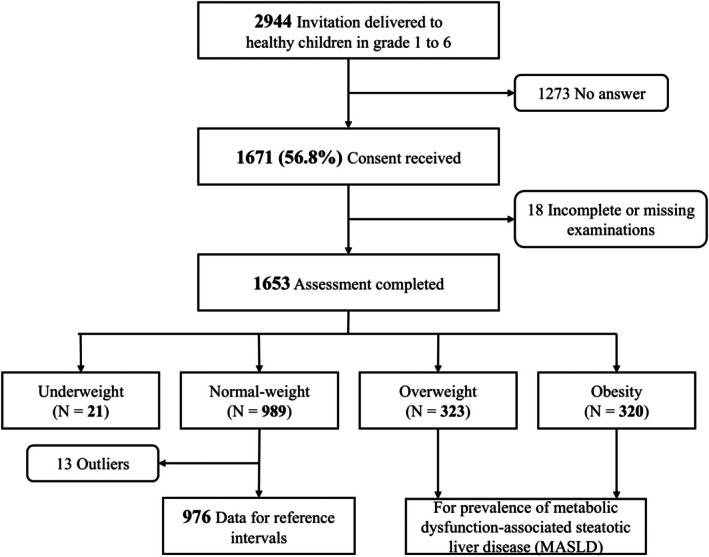
Diagram illustrating participant recruitment, inclusion and overall study structure.

Information and invitation letters were distributed to the parents or legal guardians of all students in the participating schools, with 1460 in the first wave and 1484 in the second. A total of 1671 participants consented to join the study, resulting in a response rate of 56.8%. However, 18 participants were excluded due to incomplete or missing assessments from body composition analysis or FibroScan tests. The final analysis was conducted on 1653 healthy children (778 boys and 875 girls) in school grades 1 to 6, aged 6.1–12.7 years (mean age, 9.5 ± 1.7 years). FibroScan examinations were performed using the M probe in 1622 (98%) children and the XL probe in 31 (2%). A study flowchart is provided on Figure 1.

### 3.2 Correlation Between Transient Elastography Parameters and Body Composition Analysis

Table 1 summarises the basic characteristics, FibroScan measures, and body composition estimates by BMI category. CAP values increased progressively across BMI categories from underweight to obesity (*p* < 0.001 by one‐way ANOVA, Table 1). In contrast, LSM values did not differ significantly across the four BMI categories (*p* = 0.052 by one‐way ANOVA, Table 1). All other measures showed significant differences among groups. Participants in wave one, conducted during the COVID‐19 lockdown, had significantly higher BMI *z*‐scores, lower lean mass, and higher CAP values compared to those in wave two, which was conducted after the lockdown period (Table S1).

**TABLE 1:** Participant characteristics in children with different BMI categories.

Clinical measures	Underweight (*n* = 21)	Normal (*n* = 989)	Overweight (*n* = 323)	Obesity (*n* = 320)	*p* ^a^
Age (years)	9.8 ± 1.9	9.4 ± 1.7	9.6 ± 1.7	9.8 ± 1.6	< 0.001
Sex					
Male	7 (33%)	425 (43%)	139 (43%)	207 (65%)	
Female	14 (67%)	564 (57%)	184 (57%)	113 (35%)	
Heigh (cm)	131.9 ± 11.7	133.4 ± 12.4	138.1 ± 11.9	141.9 ± 11.8	< 0.001
Weight (kg)	23.3 ± 4.9	29.6 ± 7.6	39.1 ± 9.5	51.7 ± 14.8	< 0.001
BMI (kg/m^2^)	13.2 ± 0.6	16.3 ± 1.6	20.2 ± 1.7	25.1 ± 3.7	< 0.001
Height *z*‐score	−0.9 ± 1.0	−0.2 ± 1.0	0.3 ± 0.9	0.8 ± 0.9	< 0.001
BMI *z*‐score	−2.4 ± 0.4	−0.1 ± 0.7	1.5 ± 0.3	2.8 ± 0.7	< 0.001
Controlled attenuation parameter (dB/m)	173 ± 34	181 ± 32	199 ± 36	247 ± 55	< 0.001
Liver stiffness measurement (kPa)	4.1 ± 0.7	4.3 ± 0.9	4.3 ± 0.9	4.4 ± 1.0	0.052
Body fat mass (kg)	3.5 ± 1.0	6.3 ± 2.6	12.2 ± 3.8	20.5 ± 7.7	< 0.001
Trunk fat mass (kg)	0.5 ± 0.4	2.1 ± 1.5	5.3 ± 2.1	9.8 ± 3.8	< 0.001
Percentage body fat (%)	15.1 ± 3.6	21.1 ± 5.0	30.9 ± 4.6	39.1 ± 5.0	< 0.001
Trunk percentage fat (%)	6.4 ± 4.3	15.9 ± 7.7	30.1 ± 5.8	39.3 ± 5.0	< 0.001
Free fat mass (kg)	19.8 ± 4.4	23.2 ± 5.8	26.9 ± 6.4	31.2 ± 8.1	< 0.001
Skeletal muscle mass (kg)	9.6 ± 2.6	11.7 ± 3.4	13.9 ± 3.8	16.5 ± 4.8	< 0.001

*Note:* Data were presented as Mean ± SD.

^a^ Data were compared among groups using one‐way ANOVA.

### 3.3 Derivation of Reference Values for CAP and LSM

**FIGURE 2:**
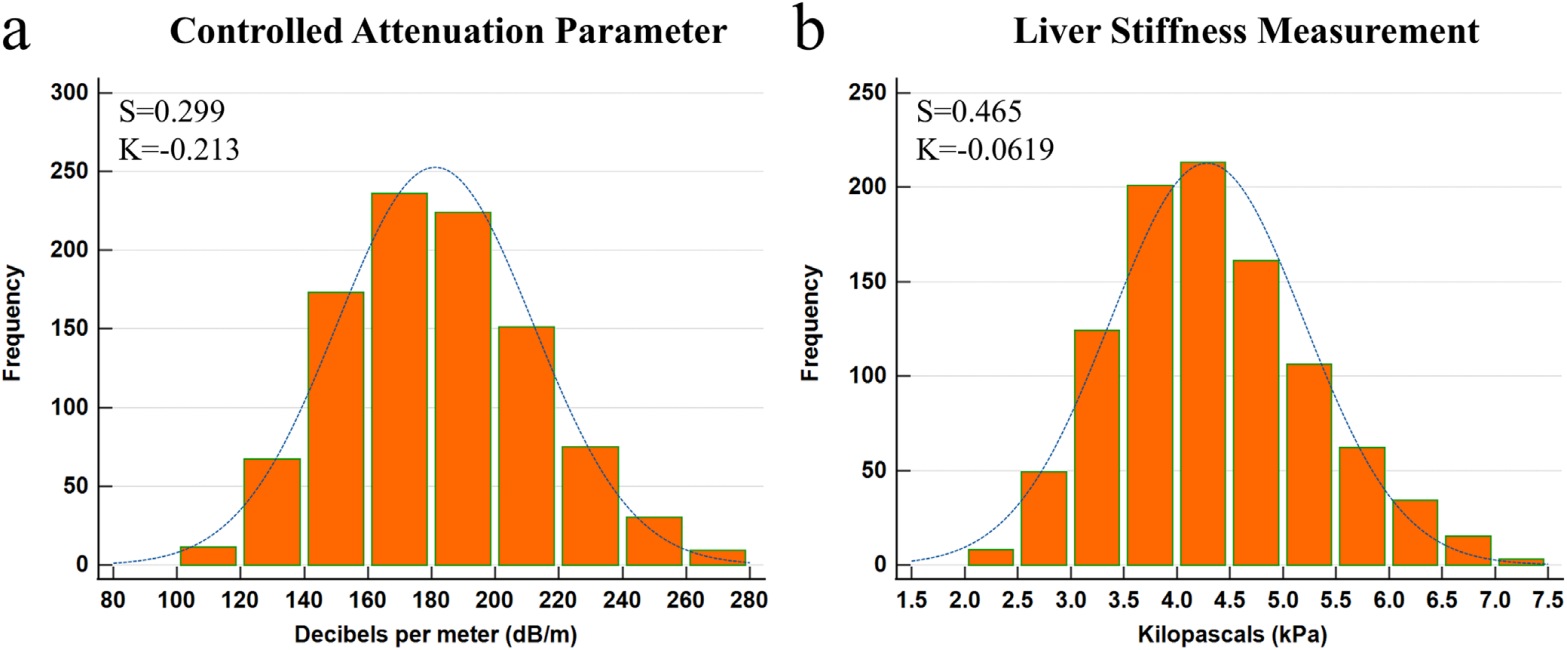
Histogram of (a) controlled attenuation parameter and (b) liver stiffness measurement in children with normal weight, showing skewness (S), kurtosis (K) and overlaid Gaussian fitting curves.

There were 989 children with normal weight. Reference intervals for CAP and LSM were established using data from 976 participants with normal weight after excluding 13 outliers identified by the Tukey test. Among these 976 participants, CAP and LSM values showed no significant sex differences (*p* = 0.206 and 0.580, respectively); however, females exhibited a higher percentage of body fat and greater fat mass than males (Table S2). Both parameters showed minimal correlations with age or other variables (*R* = −0.006–0.160, Table S3). Histograms of CAP and LSM (Figure 2) revealed right‐skewness for both measures (CAP: skewness = 0.299, *p* < 0.001; LSM: skewness = 0.465, *p* < 0.001) with near‐mesokurtic distributions (CAP: kurtosis = −0.213, *p* = 0.145; LSM: kurtosis = −0.062, *p* = 0.737, Table 2). Both distributions deviated from normality (*p* = 0.009 for CAP and *p* < 0.001 for LSM, Kolmogorov–Smirnov test, Table 2). To address this, a Box‐Cox transformation was applied to correct skewness and support reference interval analysis. Table 2 summarises four sets of CAP and LSM reference intervals, derived using parametric and non‐parametric methods, both before and after transformation. For CAP, the upper limit (97.5th percentile) was 247 dB/m across methods, except for the parametric percentile method without transformation, which yielded 242 dB/m. A similar pattern was observed for LSM, with an upper limit of 6.3 kPa, except for the parametric percentile method without transformation, which gave 6.1 kPa.

**TABLE 2:** Distribution of CAP and LSM in children with normal weight.

Measurements	CAP (dB/m)	LSM (kPa)
(a) Test for normal distribution		
Sample size	976	976
Lowest value	108	2.3
Highest value	274	7.1
Mean (95% CI)	181 (179–183)	4.3 (4.2–4.3)
Median (95% CI)	180 (177–182)	4.2 (4.1–4.2)
Coefficient of Skewness	0.299 (*p* < 0.001)	0.465 (*p* < 0.001)
Coefficient of Kurtosis	−0.213 (*p* = 0.145)	−0.0619 (*p* = 0.737)
Kolmogorov–Smirnov test	D = 0.034 (*p* = 0.009) reject normality	D = 0.066 (*p* < 0.001) reject normality
(b) Reference intervals^a^		
Parametric percentile method		
2.5th percentile (90% CI)	121 (118–124)	2.5 (2.4–2.6)
97.5th percentile (90% CI)	242 (239–244)	6.1 (6.0–6.2)
Non‐parametric percentile method		
2.5th percentile (90% CI)	126 (123–129)	2.7 (2.6–2.8)
97.5th percentile (90% CI)	247 (241–252)	6.3 (6.2–6.5)
Parametric percentile method after Cox‐Box transformation		
2.5th percentile (90% CI)	126 (124–128)	2.7 (2.7–2.8)
97.5th percentile (90% CI)	247 (243–250)	6.3 (6.2–6.4)
Non‐parametric percentile method after Cox‐Box transformation		
2.5th percentile (90% CI)	126 (113–129)	2.7 (2.6–2.8)
97.5th percentile (90% CI)	247 (241–252)	6.3 (6.2–6.5)

Abbreviations: CAP, controlled attenuation parameter; LSM, liver stiffness measurement.

^a^ Data were presented as 90th percentiles (90% CI) following the Clinical Laboratory Standardisation Institute (CLSI) Guidelines C28‐A3.

### 3.4 Prevalence of MASLD as Per CAP Cut‐Offs

Hepatic steatosis was identified using a population‐specific cut‐off of CAP > 247 dB/m. The prevalence of MASLD was 8% in children with overweight and 49% in children with obesity. Fibrosis was defined as LSM > 6.3 kPa. The prevalence of hepatic fibrosis is 0.9% in children with overweight and 4.4% in children with obesity. No statistically significant difference in LSM was observed between children with overweight and those with obesity (4.3 ± 0.9 kPa vs. 4.4 ± 1.0 kPa, respectively, *p* = 0.075, Table 1).

**FIGURE 3:**
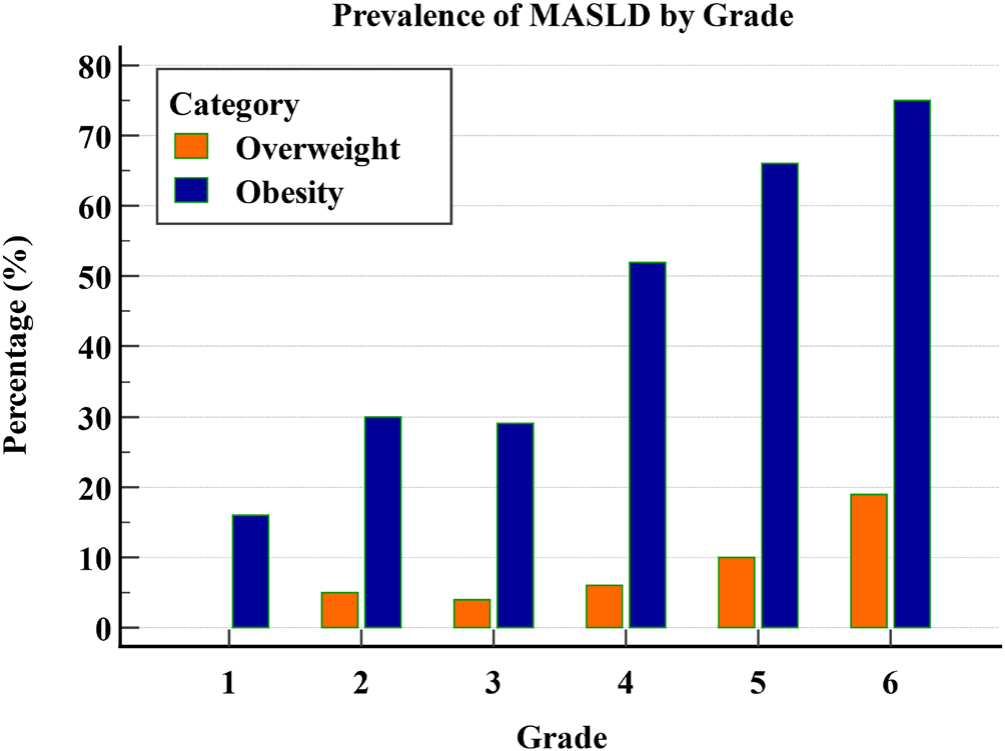
Age‐specific prevalence of metabolic dysfunction‐associated steatotic liver disease (MASLD) across different grades in primary school children with overweight and obesity.

Figure 3 shows the age‐specific prevalence of MASLD across different grades in primary school children. Among children with overweight (*n* = 323), MASLD was 0% in grade 1, fluctuating between 4% and 6% in grades 2 to 4, and gradually increased to 19% by grade 6. In children with obesity (*n* = 320), MASLD was 16% in grade 1, nearly doubled in grade 2, remained stable in grade 3, and then rose rapidly and plateaued at 75% in grade 6. Age‐specific prevalence of MASLD among children with overweight and obesity increased with increasing school grade (*p* < 0.001, Table 3).

**TABLE 3:** Prevalence of steatotic liver disease in children at school grade one to grade six.

BMI category	Grade^a^						
	1	2	3	4	5	6	*p*
Underweight	0/3 (0%)	0/3 (0%)	0/1 (0%)	0/5 (0%)	0/4 (0%)	0/5 (0%)	NA
Normal	4/161 (2%)	2/192 (1%)	5/163 (3%)	5/154 (3%)	1/164 (1%)	11/155 (7%)	0.045
Overweight	0/36 (0%)	3/64 (5%)	2/50 (4%)	3/53 (6%)	6/58 (10%)	12/62 (19%)	< 0.001
Obesity	4/25 (16%)	18/61 (30%)	12/42 (29%)	34/65 (52%)	42/64 (66%)	47/63 (75%)	< 0.001

^a^ The Cochran‐Armitage trend test was used to assess differences across children at grade 1 to 6.

## 4 Discussion

This study conducted a prospective survey to evaluate the prevalence of SLD using FibroScan in apparently healthy primary school children. The 95% reference interval for the CAP was established for healthy, normal‐weight children, with an upper limit of 247 dB/m. This finding aligns with the 2024 EASL‐EASD‐EASO Clinical Practice Guideline, which recommends a CAP cut‐off of 248 dB/m for detecting hepatic steatosis in adults. It is also consistent with previously proposed CAP thresholds for paediatric populations, which range from 225 to 277 dB/m [19, 20, 21, 22, 23]. The upper limit of LSM in this study was 6.3 kPa, corresponds well with prior studies reporting upper reference limits between 5.5 and 6.65 kPa for children [23, 24, 25].

Using population‐specific CAP thresholds, the prevalence of MASLD increased from 0% in school grade 1 to 19% in grade 6 among the 323 children with overweight while the prevalence rose significantly from 16% in school grade 1 to 75% in grade 6 among the 320 children with obesity. These findings suggest that MASLD screening for children with obesity may need to start as early as school grade 1 (approximately 6 years old), whereas for children with overweight, screening may be more appropriate by school grade 6. These results highlight distinct age‐related trends in MASLD prevalence between children with overweight and those with obesity, underscoring the importance of implementing tailored screening strategies and differentiated protocols for these two populations. Although girls with normal weight had higher fat‐related measures than boys, CAP and LSM values did not differ significantly by sex. This may reflect sex‐specific physiological differences in body composition that arise during childhood and adolescence [26], allowing girls to have greater overall fat without increased liver fat or stiffness.

MASLD is increasingly diagnosed in children; however, there is no consensus regarding the optimal age or method for screening. Guidelines differ: the North American Society for Paediatric Gastroenterology, Hepatology, and Nutrition recommends screening children with obesity and overweight at ages 9–11 [4], while the European Society for Paediatric Gastroenterology, Hepatology, and Nutrition advises screening all children with obesity, noting higher risk after age 10 [5]. The American Association for the Study of Liver Diseases offers no paediatric screening guidelines due to insufficient evidence [27]. Findings on age‐related differences remain inconsistent—some studies report a rising prevalence with age [28, 29, 30], while others, such as the study by Quirós‐Tejeira et al., found no significant differences [31], possibly due to the use of age‐ and sex‐specific ALT thresholds

The CAP has emerged as a promising tool for screening SLD in both adults and children. The 2024 Clinical Practice Guidelines from the European Association for the Study of the Liver, European Association for the Study of Diabetes, and European Association for the Study of Obesity recommend using CAP values for diagnosing SLD in adults [1]. In children, CAP has been incorporated into the diagnostic algorithm for SLD [32]. In contrast, other diagnostic tools for SLD—such as ALT and ultrasound—are either indirect or have limited sensitivity for detecting mild steatosis, while MRI, although accurate, is costly and less accessible.

Understanding age‐dependent differences is essential for determining the optimal age to initiate screening. However, most studies have focused on the overall prevalence of SLD in the general population or in children with obesity, with limited investigation into age‐related variations. Jin et al. reported a rapid increase in MASLD prevalence, detected via ultrasound, beginning at age 5 for boys and age 7 for girls with moderate to severe obesity [28]. Consistent with Jin et al., our findings support the recommendation to initiate MASLD screening for children with obesity as early as school grade 1 (approximately 6 years of age). Furthermore, our results suggest starting MASLD screening for children with overweight by school grade 6.

BMI is widely used as a surrogate measure of adiposity and, as expected, this study demonstrated that children with overweight or obesity had greater body fat and a higher percentage of body fat than their peers with normal weight. A higher BMI is also associated with elevated fasting serum insulin and leptin levels, which are linked to MASLD and may indicate earlier pubertal onset and greater height during childhood [33, 34]. However, children with overweight or obesity often exhibit a lower peak height velocity during puberty; consequently, their final adult height may not exceed that of their peers with normal weight [35, 36].

Several limitations of this study should be acknowledged. First, the CAP threshold for diagnosing MASLD was based on the 97.5th upper limit derived from healthy children rather than biopsy‐proven cases, which may have affected diagnostic accuracy. Nonetheless, the upper limit of the CAP identified in this study aligns with previous research in both adults and children. Furthermore, the inclusion of a large, community‐based sample of healthy children from a school setting, rather than a hospital‐based cohort, enhances the generalisability and reliability of the findings. Second, the recommended age for initiating MASLD screening is based on disease prevalence rather than a cost‐effectiveness assessment. Third, according to the manufacturer's manual, FibroScan probe selection for children is based on chest circumference: S1 for ≤ 45 cm, S2 for 45–75 cm, and M for > 75 cm. However, the S probes were not available during our study; therefore, only the M and XL probes were used, with probe selection between these two guided in real time by the automated probe selection tool of the FibroScan software. Fourth, this study was conducted in two waves involving different participant groups during the COVID‐19 lockdown and post‐pandemic periods. Lower lean mass, higher BMI *z*‐scores, and elevated CAP values observed in the first wave may reflect pandemic‐related lifestyle changes [37, 38]. However, as the reference intervals were calculated only in children with normal weight, the potential impact of the pandemic was likely minimised. The higher MASLD prevalence observed among children with obesity may still be partly attributable to pandemic‐related factors. Further studies are needed to clarify these effects. Additionally, this study does not address the optimal screening interval, highlighting the need for further research.

## 5 Conclusion

This study established 95% reference intervals for CAP and LSM in 976 healthy, normal‐weight children aged 6–12 years, identifying upper limits of 247 dB/m for CAP and 6.3 kPa for LSM as thresholds for diagnosing hepatic steatosis and fibrosis, respectively. Using the CAP cutoff, we determined the age‐dependent prevalence of MASLD in children with overweight and children with obesity and recommended an earlier screening initiation age for children with obesity compared to those with overweight, thereby offering a more cost‐effective and practical strategy for MASLD screening.

## Author Contributions

All authors contributed to the study conception and design. Material preparation, data collection and analysis were performed by Li‐Wen Lee, Yu‐San Liao and Chao‐Yu Chen. The first draft of the manuscript was written by Li‐Wen Lee. All authors were involved in writing the paper and had final approval of the submitted and published versions.

## Funding

This work was supported by the Ministry of Science and Technology, Taiwan (MOST 109‐2314‐B‐182A‐100) and the Chang Gung Medical Foundation (CMRPG6P0241).

## Conflicts of Interest

The authors declare no conflicts of interest.

## Supporting information


**Data S1:** Supporting Information.

## Data Availability

The data underlying this article are available in Mendeley Data, at https://doi.org/10.17632/74jbzb9d3r.1.
