# Supplementary material for: Starting Age for Screening Metabolic Dysfunction‐Associated Steatotic Liver Disease in Children Using Controlled Attenuation Parameter on Transient Elastography
Source: Pediatr Obes. 2025 Dec 22;21(1):e70080. doi: 10.1111/ijpo.70080 (PMC12722110; doi:10.1111/ijpo.70080)
Supplement: Supplementary file 1 — Data S1: Supporting Information. [file IJPO-21-e70080-s001.pdf]

Starting Age for Screening Metabolic Dysfunction-associated Steatotic Liver Disease in Children using Controlled Attenuation Parameter on Transient Elastography

Li-Wen Lee MD PhD[1,2], Jrhau Lung PhD[2,3,4], Ju-Bei Yen MD PhD[5,6], Chao-Yu Chen MD[7,8,*], Yu-San Liao MD PhD[2,9,*]

[1]Department of Diagnostic Radiology, Chang Gung Memorial Hospital, Chiayi 613016, Taiwan
[2]School of Medicine, College of Medicine, Chang Gung University, Taoyuan 333323, Taiwan
[3]Department of Medical Research and Development, Chang Gung Memorial Hospital, Chiayi 613016, Taiwan
[4]General Education Center, Chang Gung University of Science and Technology, Chiayi 613016, Taiwan
[5]Department of Pediatrics, Chang Gung Memorial Hospital, Chiayi 613016, Taiwan
[6]School of Traditional Chinese Medicine, College of Medicine, Chang Gung University, Taoyuan 333323, Taiwan
[7]Department of Obstetrics and Gynecology, Chang Gung Memorial Hospital, Chiayi 613016, Taiwan
[8]Graduate Institute of Clinical Medical Sciences, College of Medicine, Chang Gung University, Taoyuan 333323, Taiwan
[9]Department of Diagnostic Radiology, Chang Gung Memorial Hospital, Yunlin 638502, Taiwan

[*]These authors contributed equally to this work.

Contact Information

Yu-San Liao, M.D. PhD
Email address: mm601200@gmail.com
Address: Department of Diagnostic Radiology, Chang Gung Memorial Hospital, No. 1500, Gongye Road, Mailiao Township, Yunlin 638502, Taiwan
Chao-Yu Chen, M.D.
Email address: Email address: b9002031@cgmh.org.tw
Address: Department of Obstetrics and Gynecology, Chang Gung Memorial Hospital, No. 8, Sec. W., Jiapu Rd., Puzi City, Chiayi 613016, Taiwan

Supplementary Table 1. Participant characteristics in the two waves of the study

**Clinical Measures**	**First Wave**	**Second Wave**	**p-value**[a]
Sex			
Male (%)	391 (45.7%)	387 (48.5%)	
Female (%)	464 (54.3%)	411 (51.5%)	
Age, mean (SD), yrs	9.4 (1.7)	9.6 (1.7)	0.411
Height, mean (SD, cm	135.1 (12.5)	136.9 (12.7)	0.387
Weight, mean (SD), kg	35.4 (12.8)	35.8 (13.4)	0.001
BMI, mean (SD), kg/m^2	18.9 (4.1)	18.5 (4.1)	<0.001
Height z-score, mean (SD)	0 (1.0)	0.1 (1.0)	0.007
BMI z-score, mean (SD)	0.8 (1.3)	0.6 (1.4)	<0.001
CAP, mean (SD), dB/m	206 (47)	188 (43)	<0.001
LSM, mean (SD), kPa	4.5 (0.9)	4.1 (0.9)	0.225
Body fat mass, mean (SD), kg	10.4 (7.0)	9.9 (7.0)	0.035
Trunk fat mass, mean (SD), kg	4.3 (3.7)	4.1 (3.8)	0.063
Percentage body fat, mean (SD), %	27.3 (8.6)	25.4 (8.9)	0.292
Trunk percentage fat, mean (SD), %	24.0 (11.5)	22.0 (12.3)	0.295
Free fat mass, mean (SD), kg	25.0 (6.9)	25.9 (7.4)	<0.001
Skeletal muscle mass, mean (SD), kg	12.7 (4.1)	13.3 (4.4)	<0.001

Abbreviations: BMI, body mass index; CAP, controlled attenuation parameter; LSM, liver stiffness measurement.

[a] Group comparisons were performed using independent sample t-tests.

Supplementary Table 2. Basic characteristics of children with normal weight included in the calculation of 95% reference intervals for FibroScan controlled attenuation parameters and liver stiffness measurement

**Clinical Measures**	**Male (n=417)**	**Female (n=559)**	**All (n=976)**	**p value**[a]
Age, mean (SD), yrs	9.3 (1.7)	9.4 (1.7)	9.4 (1.7)	0.830
Height, mean (SD, cm	133.0 (12.1)	133.8 (12.5)	133.4 (12.3)	0.316
Weight, mean (SD), kg	29.1 (7.2)	29.9 (7.9)	29.6 (7.6)	0.089
BMI, mean (SD), kg/m^2	16.2 (1.4)	16.4 (1.6)	16.3 (1.6)	0.030
Height z-score, mean (SD)	-0.3 (0.9)	-0.2 (1.0)	-0.2 (1.0)	0.400
BMI z-score, mean (SD)	-0.2 (0.8)	-0.1 (0.7)	-0.1 (0.7)	0.184
CAP, mean (SD), dB/m	180 (31)	182 (31)	181 (31)	0.206
LSM, mean (SD), kPa	4.3 (0.9)	4.3 (0.9)	4.3 (0.9)	0.580
Body fat mass, mean (SD), kg	5.7 (2.1)	6.9 (2.7)	6.3 (2.6)	<0.001
Trunk fat mass, mean (SD), kg	1.7 (1.2)	2.4 (1.6)	2.1 (1.5)	<0.001
Percentage body fat, mean (SD), %	19.3 (4.6)	22.5 (4.8)	21.1 (5.0)	<0.001
Trunk percentage fat, mean (SD), %	13.4 (7.0)	17.7 (7.7)	15.9 (7.7)	<0.001
Free fat mass, mean (SD), kg	23.4 (5.9)	23.1 (5.7)	23.2 (5.8)	0.321
Skeletal muscle mass, mean (SD), kg	11.8 (3.5)	11.5 (3.4)	11.7 (3.4)	0.228

Abbreviations: BMI, body mass index; CAP, controlled attenuation parameter; LSM, liver stiffness measurement.

[a] Group comparisons were performed using independent sample t-tests.

Supplementary Table 3. Correlation table of parameters

-1.0 +1.0

	Height z-score	BMI z-score	Age	Body fat mass	Trunk fat mass	Percentage body fat	Trunk percentage fat	Fat free mass	Skeletal muscle mass	CAP	LSM
Height z-score	1										
BMI z-score	0.240	1									
Age	0.158	0.042	1								
Body fat mass	0.360	0.621	0.569	1							
Trunk fat mass	0.375	0.616	0.587	0.995	1						
Percentage body fat	0.039	0.626	0.079	0.781	0.758	1					
Trunk percentage fat	0.238	0.673	0.366	0.909	0.901	0.927	1				
Fat free mass	0.568	0.279	0.845	0.601	0.629	0.015	0.345	1			
Skeletal muscle mass	0.564	0.284	0.842	0.594	0.623	0.007	0.336	1.000	1		
CAP	-0.076	0.149	0.044	0.119	0.111	0.160	0.144	0.005	0.005	1	
LSM	0.012	0.050	0.042	0.016	0.016	-0.006	0.009	0.061	0.062	0.103	1

Abbreviations: CAP, controlled attenuation parameter; LSM, liver stiffness measurement
